# The Effect of Emotional Valence and Arousal on Visuo-Spatial Working Memory: Incidental Emotional Learning and Memory for Object-Location

**DOI:** 10.3389/fpsyg.2019.02587

**Published:** 2019-11-19

**Authors:** Marco Costanzi, Beatrice Cianfanelli, Daniele Saraulli, Stefano Lasaponara, Fabrizio Doricchi, Vincenzo Cestari, Clelia Rossi-Arnaud

**Affiliations:** ^1^Department of Human Sciences, LUMSA University, Rome, Italy; ^2^Department of Psychology, Sapienza University, Rome, Italy; ^3^Fondazione Santa Lucia IRCCS, Rome, Italy

**Keywords:** working memory, emotional valence, arousal, object relocation, incidental encoding

## Abstract

Remembering places in which emotional events occur is essential for individual’s survival. However, the mechanisms through which emotions modulate information processing in working memory, especially in the visuo-spatial domain, is little understood and controversial. The present research was aimed at investigating the effect of incidentally learned emotional stimuli on visuo-spatial working memory (VSWM) performance by using a modified version of the object-location task. Eight black rectangles appeared simultaneously on a computer screen; this was immediately followed by the sequential presentation of eight pictures (selected from IAPS) superimposed onto each rectangle. Pictures were selected considering the two main dimensions of emotions: *valence* and *arousal*. Immediately after presentation, participants had to relocate the rectangles in the original position as accurately as possible. In the first experiment arousal and valence were manipulated either as between-subject (Experiment 1A) or as within-subject factors (Experiment 1B and 1C). Results showed that negative pictures enhanced memory for object location only when they were presented with neutral ones within the same encoding trial. This enhancing effect of emotion on memory for object location was replicated also with positive pictures. In Experiment 2 the arousal level of negative pictures was manipulated between-subjects (high vs. low) while maintaining valence as a within-subject factor (negative vs. neutral). Objects associated with negative pictures were better relocated, independently of arousal. In Experiment 3 the role of emotional valence was further ascertained by manipulating valence as a within-subject factor (neutral vs. negative in Experiment 3A; neutral vs. positive in Experiment 3B) and maintaining similar levels of arousal among pictures. A significant effect of valence on memory for location was observed in both experiments. Finally, in Experiment 4, when positive and negative pictures were encoded in the same trial, no significant effect of valence on memory for object location was observed. Taken together results suggest that emotions enhance spatial memory performance when neutral and emotional stimuli compete with one another for access into the working memory system. In this competitive mechanism, an interplay between valence and arousal seems to be at work.

## Introduction

In our daily lives, we experience and remember many features of an event that triggers an emotional response. For instance, we well remember places where emotional experiences took place. Thus, emotional stimuli and spatial information are encoded, linked, and stored in memory for future recall.

The mechanisms through which emotions modulate long-term memory consolidation have been widely investigated (McGaugh, 2000, 2004, 2006; Phelps, 2004; Richter-Levin, 2004; Anderson et al., 2006a, b; Cestari et al., 2006; La Bar, 2007; Mather, 2007; Walker, 2010; McReynolds and McIntyre, 2012; Leventon et al., 2018). Emotional stimuli are usually classified by considering two main dimensions: *Valence*, which describes the attractiveness (positive valence) or aversiveness (negative valence) of stimuli along a continuum (negative – neutral – positive), and *arousal*, which refers to the perceived intensity of an event from very calming to highly exciting or agitating (Kensinger and Schacter, 2006). It is well established, for long-term memory, that positive and negative arousing stimuli are better remembered than neutral non-arousing ones (Canli et al., 2000; Dolcos and Cabeza, 2002; Dolcos et al., 2004; Kensinger et al., 2006; Kensinger and Schacter, 2007; Kensinger et al., 2007; La Bar, 2007; Kensinger, 2009). Moreover, long-term memory is enhanced also for neutral stimuli by increasing participants’ arousal, either through the presentation of emotional pictures (Anderson et al., 2006a; Ventura-Bort et al., 2016) or through the administration of chemical compounds usually released during emotional arousal (Buchanan and Lovallo, 2001). The latter suggests the existence of an emotional tagging which is able to increase the salience of non-emotional stimuli (Richter-Levin and Akirav, 2003).

Despite the large amount of evidence on the effect of emotion on long-term memory, the mechanisms through which emotion influence working memory are not completely understood. Contrasting results have been reported, particularly as far as the visuospatial domain is considered (Kensinger and Corkin, 2003; Shackman et al., 2006; Mather, 2007; Levens and Phelps, 2008; Mather and Nesmith, 2008; Lindstrom and Bohlin, 2011; Mather and Sutherland, 2011; Bannerman et al., 2012; Gonzalez-Garrido et al., 2015; Tavares et al., 2016).

As in long-term memory studies, the role of emotion on working memory can be investigated either by modulating the emotional content of the stimuli used in the memory task or by manipulating the mood of participants.

Manipulations of participants’ mood are usually aimed at enhancing the arousal level before performing working memory tasks. These manipulations include the presentation of affective video clips, the administration of stress-related compounds, or the exposure to threatening conditions. Overall, studies on the effect of emotional arousal on working memory performance have produced mixed results. Further, interpreting results of experiments which have manipulated emotional arousal and mood is not always straightforward. In particular, problems of internal validity (i.e., difficulties in monitoring whether the adopted manipulation produced the desired effect on arousal or mood), difficulties in monitoring whether the effects of manipulation lasted throughout the memory task, and difficulties in determining on which phase of the task the manipulation is effectively acting (e.g., encoding or retrieval) have been reported (see Mather, 2007; Moran, 2016 for reviews).

Another procedure that has been effectively used to investigate how emotional information is processed in working memory consists in manipulating the emotional content of the stimuli used in the memory task (Mather, 2007; Pessoa, 2008, 2009; Kensinger, 2009; Mather and Sutherland, 2011). Using a modified version of the Corsi-block task, in which spatial positions were signaled by emotional or non-emotional stimuli (schematic faces, real faces or pictures), Bannerman et al. (2012) found that emotions did not affect spatial working memory performance, although emotional stimuli were able to capture attention more effectively than neutral ones (Bannerman et al., 2012). Differently, in a study in which participants had to remember spatial sequences of six facial (happy and neutral) and non-facial stimuli in reverse order, a performance improvement was observed in trials in which happy faces appeared (Gonzalez-Garrido et al., 2015). However, the type of stimuli used in the latter study (faces), the length of the task and the repeated presentation of the stimuli make it difficult to rule out the involvement of a competition mechanism between emotional and non-emotional stimuli for access to working memory (an aspect which we will further discuss later on).

In another study, a negative impact of emotions on working memory performance was observed (Yoon et al., 2016). The latter used a visual working memory test and participants were instructed to maintain (forward trials) or reverse (backward trials) the order of four emotional or four neutral pictures. Furthermore, a distraction effect exerted by emotional stimuli was observed in a digit recognition task with a high working memory load, suggesting that memory reduction could be induced by a depletion of attentional resources exerted by the emotional material (Tavares et al., 2016).

In the above-mentioned studies, emotional and non-emotional stimuli were presented in separate trials of the task and conclusions regarding the effect of emotion on working memory performance were drawn by comparing the performance achieved in trials in which only emotionally valenced stimuli were presented with the performance achieved in trials in which only neutral stimuli were presented. Therefore, if one considers the emotional valence of stimuli administered during the working memory task, participants processed only one type (negative, positive, or neutral) of stimulus at a time.

On the other hand, when emotional and neutral stimuli are presented within the same encoding trial, a positive impact of emotion on memory performance always emerges. In an incidental encoding task in which both emotionally arousing and non-arousing pictures, selected from the International Affective Picture System (IAPS), appeared in different locations of the screen within the same encoding trial, participants better recognized the position of emotionally arousing pictures than the position of non-arousing ones. Interestingly, the performance improvement for emotionally arousing pictures was independent of their valence (negative or positive) (Mather and Nesmith, 2008). Therefore, the authors suggested that emotionally arousing stimuli had a priority access to the working memory system (Mather and Nesmith, 2008; Mather and Sutherland, 2011). In the same vein, Schmidt et al. (2011), by using a spatial and temporal recognition task in which mixed lists of both arousing and non-arousing emotional pictures were presented, found that memory for spatial location and temporal order for high-arousing stimuli was increased regardless of their valence (Schmidt et al., 2011). More recently, Schümann et al. (2018) found that memory recognition for emotional pictures was higher than memory for neutral pictures immediately after encoding a mixed list of positive, negative and neutral pictures (Schümann et al., 2018). Overall, the latter results are consistent with the Arousal-biased competition (ABC) theory, which predicts that encoding of within-object characteristics, like spatial location, is enhanced for arousing stimuli (Mather and Sutherland, 2011).

However, aside from arousal levels, the hypothesis that emotional valence is also important in modulating working memory performance has recently been discussed (Adelman and Estes, 2013; Kang et al., 2014; Wilson et al., 2016). In particular, an effect of valence on visuo-spatial working memory (VSWM) performance has been suggested in a recognition task in which memory for dot locations was increased by presenting negative pictures on the same side of the screen in which dots appeared (Wilson et al., 2016).

Putman et al. (2004) investigated the effect of valence on spatial working and long-term memory in healthy young women by using a face relocation task, in which both emotional (happy and fearful) and non-emotional faces were simultaneously encoded. Happy faces were better relocated than neutral ones in the immediate test, while both negative and happy faces were better relocated than neutral ones in the long-term memory test (Putman et al., 2004). These results suggest a valence-specific effect for the immediate memory performance, but not for the memory performance at long-term (Putman et al., 2004).

Considering the literature reported above, it is possible to hypothesize that emotions increase spatial memory when neutral and emotional stimuli are presented within the same encoding trial, suggesting the existence of a competition mechanism. Further, the role played by arousal and valence in regulating the competitive access to the working memory system is not clear. These two aspects deserve investigation.

In the present study, we sought (a) to verify the competition hypothesis and (b) to ascertain the effect of valence and arousal in determining access to VSWM. To this purpose, the effect of incidentally learned emotional stimuli on VSWM performance was investigated. We used a modified version of the object-relocation task in which participants had to encode and remember the position of eight black rectangles simultaneously presented. While the rectangles were on the screen, emotional and non-emotional pictures were presented sequentially and overlapped for a brief period of time the rectangles. The ability to relocate the objects was tested immediately after presentation while the incidental learning of the emotional or non-emotional pictures was evaluated 24 h after the visuo-spatial memory test, using a free recall test. In Experiment 1A, the pictures associated to the rectangles during encoding were either all negative or all neutral (no competition), while in Experiment 1B, half of the rectangles were accompanied by negative pictures while the other half by neutral pictures (competition). Following the competition hypothesis, we expected an effect of emotion only in the second experiment. Similarly, Experiment 1C was performed by associating neutral and positive pictures (the latter of the same arousal level as negative pictures used in the previous experiment) to the rectangles, in order to explore competition mechanisms also with positive valenced stimuli. Further experiments were performed to better clarify which emotional feature affects spatial working memory performance in this paradigm. More specifically, in Experiment 2 we examined the effect of arousal by comparing the level of performance, under competition conditions i.e., with both negative and neutral pictures being presented in the same trial, with either low or high arousal pictures. Since in all previous experiments emotional and neutral pictures used within the same trial to elicit competition differed in both valence and arousal, we performed a further experiment in which we manipulated the valence of pictures, keeping the arousal level constant. To this purpose, in Experiment 3A we presented negative and neutral pictures with similar low levels of arousal, and in Experiment 3B positive and neutral pictures again with similar low levels of arousal. Since all the experiments performed to examine competition entailed the presentation of emotional versus neutral stimuli within the same trial, we designed a last experiment in order to verify the competition hypothesis when emotional stimuli with different valence (positive and negative), but similar arousal levels, compete with one another within the same trial (Experiment 4).

## Materials and Methods

### Participants

A total of 226 (154 females; age: 23.24 ± 4.19) undergraduate students in Psychology at LUMSA and Sapienza Universities voluntarily participated in the experiments. This study was carried out in accordance with the recommendations of Committees for Ethics, Department of Psychology Sapienza, University of Rome, and LUMSA University. All subjects gave written informed consent in accordance with the Declaration of Helsinki. All participants were Italian speakers and reported having normal or corrected-to-normal vision.

### Materials and Procedures

**FIGURE 1:**
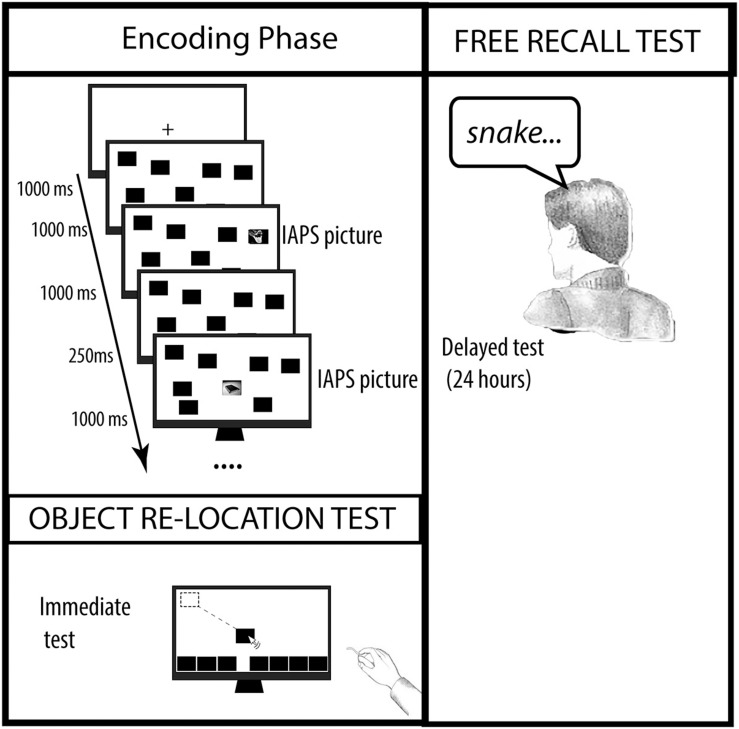
A schematic representation of the experimental procedure used in all experiments.

To study the effect of the incidental presentation of emotional stimuli on VSWM performance, a modified version of the object relocation task (Kessels et al., 1999) was administered (see Figure 1). Participants sat in front of a PC screen and an instruction slide was shown in which they received information about the object-relocation task, but not about the presentation of emotional pictures.

Thirty-six pictures were selected from the International Affective Picture System (IAPS; Lang et al., 2008). Mean valence and arousal values for the pictures used in each experiment are reported in Table 1, and IAPS codes in the Appendix.

**TABLE 1:** Valence and arousal values of IAPS pictures used in the experiments.

	**Pictures (*n*)**	**Valence (mean ± SD)**		**Arousal (mean ± SD)**	
Exp. 1A	Negative (8) Neutral (8)	3.12 ± 0.26 4.69 ± 0.43	*t*(14) = −8.912; *p* < 0.0001	5.7 ± 0.17 2.6 ± 0.25	*t*(14) = 28.496; *p* < 0.0001
Exp. 1B	Negative (4) Neutral (4)	3.10 ± 0.26 4.69 ± 0.25	*t*(6) = −8.85; *p* < 0.0001	5.7 ± 0.25 2.5 ± 0.17	*t*(6) = 21.34; *p* < 0.0001
Exp. 1C	Positive (4) Neutral (4)	7.73 ± 0.31 4.69 ± 0.25	*t*(6) = 9.12; *p* < 0.0001	5.76 ± 0.37 2.5 ± 0.17	*t*(6) = 16.03; *p* < 0.0001
Exp. 2	Negative – High arousal (4) Negative – Low arousal (4) Neutral – High arousal (4) Neutral – Low arousal (4)	2.55 ± 0.77 3.1 ± 0.26 4.91 ± 0.04 4.7 ± 0.25	*F*(3,12) = 30.42; *p* < 0.0001 *p* < 0.0001 Neg vs. Neutral	7.12 ± 0.19 5.7 ± 0.25 2.02 ± 0.32 2.47 ± 0.17	*F*(2,12) = 4.13; *p* < 0.0001 *P* < 0.0001 Neg-Low vs. Neg-High *P* < 0.0001 Neg vs. Neutral
Exp. 3A	Negative (4) Neutral (4)	3.12 ± 0.72 4.72 ± 0.33	*t*(6) = 4.02; p < 0.01	3.81 ± 0.21 3.72 ± 0.18	*t*(6) = 0.64; *p* = n.s.
Exp. 3B	Positive (4) Neutral (4)	7.85 ± 0.15 4.72 ± 0.33	*t*(6) = −17.13; *p* < 0.01	3.81 ± 0.54 3.72 ± 0.18	*t*(6) = −2.28; *p* = n.s.
Exp. 4	Negative (4) Positive (4)	3.10 ± 0.26 7.96 ± 0.39	*t*(6) = −20.93; *p* < 0.0001	5.7 ± 0.25 5.68 ± 0.33	*t*(6) = 0.09; *p* = n.s.

*Range of all IAPS pictures (*n* = 1194) valence from 1.31 to 8.34; arousal from 1.72 to 7.35. Range IAPS pictures used in the present experiments (*n* = 36) valence from 1.62 to 8.34; arousal from 1.72 to 7.35.*

When participants felt ready, they pressed a button to begin the encoding phase which was signaled by the presentation of a cross in the center of the screen for 1000 ms. Immediately after cross presentation, eight black rectangles (170 × 128 px; 72 dpi) simultaneously appeared in random positions of the screen. After 1000 ms, eight pictures selected from IAPS appeared one at a time superimposed on each rectangle. Each picture was presented for 1000 ms (ISI: 250 ms). Thus, the encoding phase lasted 11 s and 750 ms. The test phase took place immediately after the end of the encoding phase. All the black rectangles appeared at the bottom of the screen and participants had to relocate them as accurately as possible, using the mouse. Memory for object location was evaluated considering the distance between the original position and the closest relocated object. Long-term memory for incidentally learned pictures was evaluated 24 h later by a free recall task: participants were asked to verbally recall, by speaking the name of the objects depicted in the pictures they had seen on the previous day. After the free recall test, pictures were again presented one at a time, for 7 s, on the screen. Participants were instructed to view each picture and to subjectively evaluate valence and arousal of each picture by using the Self-Assessment Manikin (SAM).

#### Experiment 1

##### Experiment 1A

In Experiment 1A object positions were tagged by presenting negative pictures to one group (Negative group) and neutral pictures to a second group (Neutral group), in a between-subject manipulation. Therefore, the effect of emotional learning on VSWM was investigated in this first experiment in a “non-competitive” fashion. In brief, participants (32 females and 8 males; age:23.32 ± 4.25) were randomly assigned to two different groups: (i) *Negative* group in which object positions were tagged by negative IAPS pictures; (ii) *Neutral* group in which object positions were tagged by neutral IAPS pictures.

Since in this experiment a between- subject design was used, to further investigate if performance would be affected by basal differences in motor and spatial abilities of participants we performed a supplemental experiment in which a control condition followed the experimental trial with IAPS picture. In this control condition, the procedure was identical to that previously described but the images associated to rectangles were built by scrambling pixels of different colors.

##### Experiment 1B

In Experiment 1B, within a single trial, half of the object positions were tagged by the presentation of neutral pictures and the other half by negative pictures, thus allowing a within-subject manipulation. Therefore, the effect of emotional learning on VSWM was investigated in a “competitive” fashion. In brief, after participants (20 females and 8 males; age: 23.18 ± 2.43) simultaneously watched all eight objects for 1000 ms, four negative and four neutral pictures appeared superimposed on each object. The starting position of picture presentation was varied and each of the eight rectangles on the display could act as the starting position, thus yielding eight different configurations. Further, for half of the participants the first item presented was an emotional picture (negative in Experiments 2, 3, and 5; positive in Experiment 4), followed by a neutral one, while for the other half, presentation started with a neutral picture followed by an emotional one, resulting in 16 different configurations. The order was counterbalanced across participants.

##### Experiment 1C

The third experiment was aimed at investigating the effect of emotional learning on VSWM in a “competitive” fashion, like in the second experiment, but object positions were tagged by the presentation of either positive or neutral pictures within a single trial. In brief, after participants (19 females and 6 males; age: 24.4 ± 3.58) simultaneously watched all eight objects for 1000 ms, four positive and four neutral pictures appeared superimposed on each object. The order of picture presentation was randomized like in Experiment 2.

#### Experiment 2

The second experiment was aimed at investigating whether the increase in picture’s arousal enhanced VSWM performance. Participants (30 females and 19 males; age: 23.64 ± 4.29) were submitted to the object relocation task following the same procedure as in the previous experiment 1B. They were randomly assigned to two different groups: (i) *High arousal* in which object positions were tagged by four negative pictures with high arousal values, and four neutral pictures; (ii) *Low arousal* in which object positions were tagged by four negative pictures with low arousal values, and four neutral pictures.

#### Experiment 3

##### Experiment 3A

The present experiment was designed to investigate the effect of emotional valence on VSWM performance. Participants (11 females and 9 males; age: 21.2 ± 2.85) were submitted to the object relocation task following the same procedure as in the Experiment 1B, but object positions were tagged by the presentation of four negative and four neutral pictures with similar arousal values.

##### Experiment 3B

Participants (17 females and 4 males; age: 23.4 ± 2.79) were submitted to the object relocation task following the same procedure as previously, but object positions were tagged by the presentation of four positive and four neutral pictures with similar arousal values.

#### Experiment 4

The last experiment was designed to further investigate the effect of emotional valence on VSWM performance. Eighteen participants (13 females; age: 23.6 ± 2.15) were submitted to the object relocation task. The procedure was identical to the one used previously but the pictures differed in order to have four negative and four neutral pictures with similar levels of arousal.

### Data Analysis

In all experiments the displacement error was calculated as the distance (expressed in pixel) between the center of the originally positioned object and the center of the closest relocated object. For the evaluation of picture’s memory, the proportion of correctly recalled pictures was considered for statistical analyses.

Student’s *t*-test and two-way ANOVAs were performed when appropriate on displacement error and memory recall as well as on the level of arousal and valence of pictures. All statistical analyses were performed with SPSS 24 and considering alpha = 0.05.

## Results

### Emotional Stimuli Affect Visuospatial Working Memory When They Are in Competition With Neutral Stimuli

The first experiments were carried out in order to verify the competition hypothesis.

**FIGURE 2:**
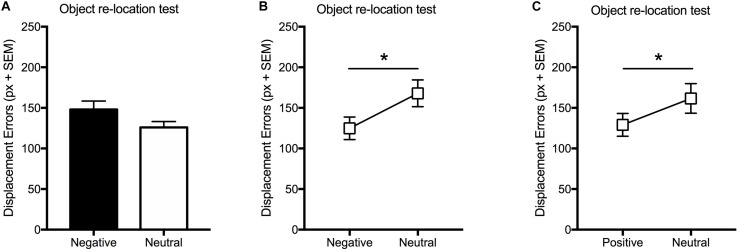
Effect of emotional stimuli on VSWM when neutral and emotional information is not **(A)** or is **(B,C)** presented within the same encoding trial. Mean displacement error (pixel) displayed by participants in the immediate re-location test **(A)** when neutral and negative information is not presented within the same encoding trial, **(B)** when neutral and negative information is presented within the same encoding trial, and **(C)** when neutral and positive information is presented within the same encoding trial. Bars: standard error mean; ^∗^*p* < 0.05.

In Experiment 1A object-positions were tagged either by neutral or negative pictures. Thus, the effect of emotion on VSWM was investigated as a between-subject factor. Statistical analysis (unpaired *t*-test) showed no differences [*t*(38) = 1.73; *p* > 0.05] in the relocation performance between groups (Figure 2A). Memory for incidentally learned pictures was evaluated in a free recall test carried out 24 h after the object-relocation task. Statistical analysis (unpaired *t*-test) revealed that the number of pictures recalled was significantly [*t*(38) = 2.58; *p* < 0.05) greater for negative than for neutral pictures (Table 2). In order to investigate whether a confounding effect of the individual differences in motor control or in spatial working memory ability could influence relocation performance, we carried out a supplemental experiment in which 3 h after the main task performed with negative and neutral IAPS pictures (IAPS pictures condition), participants performed a further object relocation task in which object-positions were tagged by the presentation of pictures built by scrambling pixels of different colors (Control condition). Results were analyzed by means of a two-way ANOVA considering a Group factor with two levels, Negative and Neutral (i.e., participants who were administered negative or neutral IAPS pictures in the first encoding condition), and a Condition factor with two levels, IAPS pictures or Control scrambled pictures. The analysis revealed no significant effects of Group [Negative vs. Neutral; *F*(1,23) = 0.11; *p* = n.s.], of Condition [IAPS pictures vs. Control pictures; *F*(1,23) = 1.62; *p* = n.s.] and no interaction between factors [*F*(1,23) = 0.35; *p* = n.s.; Supplementary Figure S1].

**TABLE 2:** Proportion of remembered pictures.

	**Mean (±SEM)**	
Experiment 1A (*n* = 20 + 20)	Negative 0.200 (0.030)	Neutral 0.096 (0.032)
Experiment 1B (*n* = 28)	Negative 0.232 (0.048)	Neutral 0.116 (0.033)
Experiment 1C (*n* = 25)	Positive 0.270 (0.043)	Neutral 0.140 (0.029)
Experiment 2 (*n* = 29)	Negative HIGH arousal 0.647 (0.052)	Negative LOW arousal 0.425 (0.063)
Experiment 2 (*n* = 20)	Neutral 0.405 (0.042)	Neutral 0.188 (0.051)
Experiment 4 (*n* = 20)	Negative 0.225 (0.040)	Positive 0.238 (0.046)

In Experiment 1B, object positions during the encoding phase were tagged by the incidental presentation of both neutral and negative pictures, which appeared one at time. Thus, the effect of emotion on VSWM was investigated as a within-subject factor. Statistical analysis (paired *t*-test) carried out on the displacement errors revealed a significant effect [*t*(27) = −2.37; *p* < 0.05] of emotion, with negative-tagged objects being better relocated than neutral-tagged objects (Figure 2B). Like in Experiment 1, the number of remembered pictures in the delayed free recall test was significantly [*t*(27) = 2.22; *p* < 0.05] greater for negative than for neutral pictures (Table 2).

In Experiment 1C, object positions during encoding phase were tagged by the incidental presentation of both neutral and positive pictures, which appeared one at time. Thus, the effect of emotion on VSWM was investigated as a within-subject factor. Statistical analysis (paired *t*-test) carried out on the displacement errors revealed objects associated to positive pictures were better relocated than those associated to neutral ones [*t*(24) = **−**2.45; *p* < 0.05] (Figure 2C). The number of remembered pictures in the delayed free recall test was higher for positive pictures than for neutral ones [*t*(24) = 2.59; *p* < 0.05] (Table 2).

These results confirm the hypothesis that emotional information enhances spatial memory performance when emotional and non-emotional stimuli compete with one another for accessing the working memory system (competition effect). Moreover, the emotional content of images increased long-term memory for the incidentally learned pictures. Since negative and positive pictures were more activating than neutral ones, a possible effect of emotional arousal can be envisaged which allows emotional pictures to get a priority access to the memory system (Mather and Sutherland, 2011). However, since negative and positive pictures are also emotionally valenced, in comparison to neutral ones, a possible role of valence in determining a priority access to the memory system cannot be ruled out. In this case, it is possible to hypothesize a “dichotomic system” in which competition is driven by what is endowed with an emotional valence (either negative or positive) and what is not (neutral). The following experiments are planned to verify the role of “arousal” and “valence” in the emotion-enhancing spatial memory performance in competition condition.

### Increasing Arousal of Negative Pictures Did Not Enhance Spatial Working Memory Performance in the Competition Condition

Experiment 2 was specifically aimed at investigating the role of emotional arousal in the competition mechanism. The ABC theory predicts that the level of arousal of emotional stimuli regulates the access to the working memory system, enhancing the encoding of within-object characteristics, like spatial position (Mather and Sutherland, 2011). Therefore, we expected that increasing the arousal level of negative pictures would lead to an enhancement of spatial working memory performance. In the high-arousal group, rectangles were tagged by the presentation of neutral and high-arousal negative pictures while in the low-arousal group, rectangles were tagged by neutral and negative pictures with low arousal values (Table 1).

**FIGURE 3:**
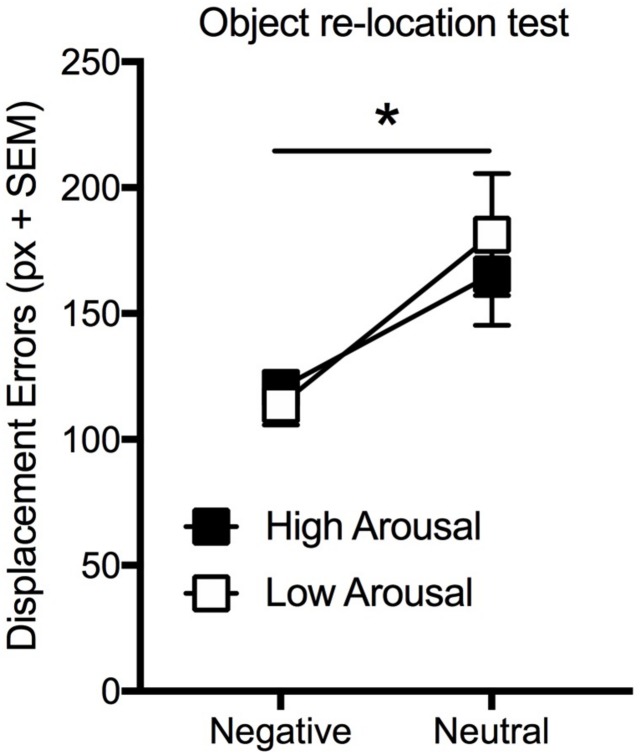
Effect of arousal (High and Low Negative vs. Neutral) on VSWM. Mean displacement error (pixel) displayed by participants in the immediate re-location test. Bars: standard error mean; ^∗^*p* < 0.05.

Statistical analysis (two-way ANOVA) carried out on the displacement errors considering arousal (high vs. low) as between factor and valence (negative vs. neutral) as within factor revealed that the effect of valence was significant [*F*(1,47) = 15.71; *p* < 0.01] whereas neither the effect of arousal [*F*(1,47) = 0.06; *p* = 0.62] nor the interaction [*F*(1,47) = 0.62; *p* = 0.43] were significant (Figure 3). Interestingly, in the free recall test, the number of both neutral and negative pictures correctly recalled was significantly greater for the high-arousal group than for the low-arousal one [Two-way ANOVA: arousal effect: *F*(1,47) = 13.88, *p* < 0.005; valence effect: *F*(1,47) = 28.38, *p* < 0.005; interaction effect: *F*(1,47) = 0.002, *p* = 0.96] (Table 2).

These results indicate that arousal manipulation, obtained by selecting negative pictures with different level of arousal (high vs. low), did not lead to a significant enhancement in spatial working memory performance, whereas it did enhance long-term memory for both negative and neutral pictures.

Since arousal did not seem to significantly impact on the competition between negative and neutral information for accessing the working memory system, we explored the possible effect of valence in this competition.

### Valence Affects Spatial Working Memory When Arousal Is Kept Constant in Competition Condition

In Experiment 3, we sought to better ascertain the role of valence in spatial working memory by keeping the level of pictures’ arousal constant, and by modulating the level of pictures’ valence. Thus, in Experiment 3A we tagged the object position with negative and neutral pictures but this time pictures had comparable arousal levels (Table 1). In Experiment 3B, rectangle position was tagged by positive and neutral pictures with comparable arousal values (Table 1). In both cases valence is a within-subject factor.

**FIGURE 4:**
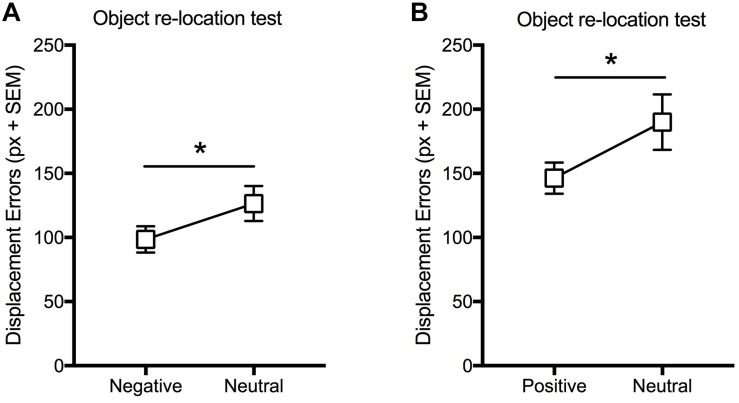
Effect of valence (Negative or Positive vs. Neutral) on VSWM. Mean displacement error (pixel) displayed by participants in the immediate object re-location test **(A)** when neutral and negative information with similar level of arousal is presented within the same encoding trial, and **(B)** when neutral and positive information with similar level of arousal is presented within the same encoding trial. Bars: standard error mean; ^∗^*p* < 0.05.

In Experiment 3A, the statistical analysis (paired *t*-test) carried out on displacement errors (Figure 4A) revealed a significant effect of valence [*t*(17) = −2.89; *p* < 0.05] with lower displacement errors for negative-related objects.

In Experiment 3B, the statistical analysis (paired *t*-test) carried out on displacement errors (Figure 4B) revealed that objects associated to positive pictures were better relocated than those associated to neutral ones [*t*(20) = 2.28; *p* < 0.05] (Figure 4B).

The results of these experiments indicate that when arousal is kept constant between neutral and negative pictures, or between positive and neutral pictures, valence significantly affects visuo-spatial performance.

### The Effect of Emotion on Working Memory Performance Vanishes When all Stimuli Have an Emotional Value

**FIGURE 5:**
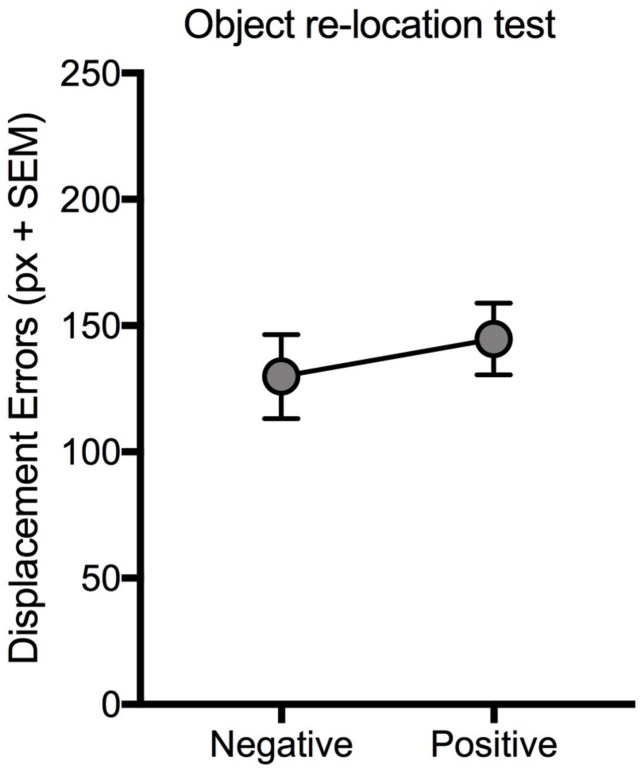
Effect of valence (Positive vs. Negative) on VSWM. Mean displacement error (pixel) displayed by participants in the immediate object re-location test when negative and positive information with similar level of arousal is presented within the same encoding trial. Bars: standard error mean.

Experiment 4 was designed in order to verify the competition hypothesis when neutral stimuli are not presented, and competition within the same trial is only among emotional pictures with different valence (positive and negative) values. Thus, object positions were tagged by negative and positive pictures, with comparable arousal levels (Table 1). Statistical analyses (paired *t*) carried out on displacement errors (Figure 5) and on long-term memory (Table 2) revealed no significant effects of valence [*t*(19) = −0.7; *p* > 0.05 and *t*(19) = 0.24; *p* > 0.05, respectively]. Interestingly, the displacement error of both positive- and negative-related objects (144.74 ± 14.18 and 129.84 ± 16.65) in this experiment was similar to displacement error of neutral-related objects (125.95 ± 9.04) in Experiment 1A, i.e., in absence of competition [one-way ANOVA; *F*(2,57) = 0.56; *p* = 0.58], suggesting that when positive and negative stimuli compete for accessing the working memory the “competition effect” disappears.

In the delayed free recall test, both positive and negative pictures of this experiment were better remembered (0.23 ± 0.04 and 0.24 ± 0.05) than neutral pictures (0.12 ± 0.03) of Experiment 1B [one-way ANOVA; *F*(2,65) = 3.2; *p* < 0.05]. Notably, in this case, both positive and negative pictures had a higher arousal compared to the neutral pictures of Experiment 1B.

Taken together the results of this experiment indicate that when competition occurs among stimuli that all have an emotional value (either positive or negative), the effect of emotion on working memory performance vanishes.

## Discussion

The present study was aimed at investigating the effect of the incidental presentation of emotional stimuli on VSWM. To this purpose an object-relocation task was used in which emotional stimuli appeared superimposed on the objects to be relocated.

The first hypothesis tested was that the emotional content of stimuli affects memory for object position only when neutral and emotional stimuli are in competition with one another, i.e., when they are presented within the same encoding trial. Previous studies on the effect of emotion on spatial working memory have yielded contrasting results: positive, negative or no effects of processing stimuli with an emotional content on spatial working memory performance have been reported (Putman et al., 2004; Dolcos and McCarthy, 2006; Mather et al., 2006; Mather and Nesmith, 2008; Schmidt et al., 2011; Bannerman et al., 2012; Gonzalez-Garrido et al., 2015). For example, there is evidence showing that emotions have a negative impact on spatial working memory because the emotional content of stimuli captures attention, subtracting resources for processing other within-object features, like spatial location (Mather et al., 2006). The possibility that the attentional bias exerted by emotional stimuli distracts from the main task, thus impairing memory performance, has been suggested (Dolcos and McCarthy, 2006; Bannerman et al., 2010; Iordan et al., 2013; Tavares et al., 2016). Although emotional stimuli are able to capture attention, there are studies showing no effect or even an improving effect of emotion on spatial working memory performance (Bannerman et al., 2012; Gonzalez-Garrido et al., 2015). Bannerman et al. (2012) found no effect of emotions on spatial working memory evaluated with a Corsi-Block Task in which spatial positions were highlighted by the presentation of emotional pictures. The authors explained the lack of effect by considering the possibility that the attentional bias exerted by the emotional content of stimuli is specifically directed to object-identity recognition (i.e., what the stimulus is). Therefore, since “what” the stimulus is and “where” the stimulus is located are encoded by different processes, spatial memory could not be affected by the emotional content of stimuli (Bannerman et al., 2012). Gonzalez-Garrido et al. (2015) recorded EEG during a face relocation task and found that spatial memory for happy face position, as well as the electrophysiological component linked to the attentional process, is increased for emotional faces. The authors suggested that emotional faces attracted more attention increasing spatial memory performance through a domain-general attention-based mechanism (Gonzalez-Garrido et al., 2015).

In the present study, the results of the first set of experiments seem to indicate that the emotional content of pictures enhanced VSWM only when emotional and neutral information was processed within the same trial, suggesting the existence of a “competition effect.” Although the interpretation of our results must be modulated by the fact that Experiment 1A entailed a between group comparison with a low level of power, it appears that, if both neutral and negative stimuli have been encountered in the environment (like in Experiment 1B), then the position of the latter is better remembered. Instead, if stimuli encountered all had a similar emotional impact (like in our Experiment 1A), the enhancing effect on spatial working memory performance, observed in the competition condition, disappears. The enhancing effect of emotion on spatial working memory performance in competition condition has been replicated also with neutral and positive stimuli (Experiment 1C).

These results seem to be in line with the ABC theory (Mather and Sutherland, 2011). According to the latter, the arousal level of emotional stimuli should increase the strength of mental representations for emotional material at the expense of the non-emotional one, through an “ABC” process. This “ABC” begins with perception, increasing the perceptual capability for binding features (such as object location) when stimulus competition occurs. The advantage yielded by arousing stimuli is maintained in the working memory system, where emotionally tagged information dominates the competition for mental resources (Richter-Levin and Akirav, 2003; Mather and Sutherland, 2011; Lee et al., 2014; Dunsmoor et al., 2015; Hur et al., 2017; Schweizer et al., 2019). The advantage exerted by emotion in “competition” condition could explain the improving effect of the emotional content of stimuli on spatial working memory performance observed in Experiments 1B and 1C.

In the absence of competition, when all stimuli have a similar emotionally arousing content, like in Experiment 1A, it is plausible to hypothesize that the emotional content of stimuli captures the attentional resources, facilitating the encoding of within-object features. However, when competition between the mental representation of emotional and non-emotional stimuli is lacking (due to the fact that all the stimuli have a similar emotional impact), no prioritization effect emerges during stimulus processing in working memory. This could explain why emotion did not affect spatial working memory performance in Experiment 1A.

To the best of our knowledge, this is the first time that the competition hypothesis is evaluated by comparing the results of two experimental manipulations in which the emotional stimuli compete or not for accessing to working memory. The results of our experiments indicate that competition among stimuli is an important factor to consider when the effect of emotion on memory performance is investigated. Having ascertained that competition is necessary in order for emotions to influence VSWM performance, the question arises as to which dimension, arousal and/or valence, is mainly involved in modulating working memory performance.

In Experiment 2 we sought to verify the prediction that arousal is the emotional dimension that mainly determines the enhancement in spatial working memory performance for the relocation of negative-related objects. A better location memory has been found for arousing pictures independently of their valence, suggesting that arousal, rather than valence, is the critical dimension for the emotion-enhancing memory effect (Mather and Nesmith, 2008). Therefore, we hypothesized that increasing the arousal level of negative pictures should lead to an improvement in spatial memory performance. Overlapping performances in relocating objects associated with high- and low-arousing pictures were observed (Experiment 2), although both high and low arousing negative-related objects were better relocated than neutral-related objects. Even if we cannot definitely rule out the possibility that this pattern of results is linked to a floor effect, our findings do not seem to be in line with the prediction that arousal is the main emotional dimension affecting VSWM. On the other hand, like for previous experiments, we observed a better memory performance for negative pictures than for neutral ones in the delayed (24 h) free recall task. Moreover, in the second experiment we found that high arousing negative pictures were better remembered than low arousing ones. The latter results support the notion that arousal is mainly involved in long-term memory formation (Bradley et al., 1992; McGaugh, 2000; LaLumiere et al., 2017). Interestingly, the memory improvement effect exerted by the *high arousal condition* was extended also to neutral pictures: neutral pictures presented together with high arousing negative pictures were better remembered than neutral pictures encoded together with low arousing negative pictures.

Two distinct neuronal pathways in the brain have recently been observed for the processing of arousal and valence. Arousing information is processed by an amygdala – hippocampus circuit, while valenced non-arousing information is processed by a PFC-hippocampus circuit, indicating two separate routes for arousal and valence (Kensinger and Corkin, 2004). The former reflects a relatively automatic effect of emotion on long-term memory (especially on memory consolidation), while the latter is associated with a controlled encoding process which supports working memory processes (such as, elaboration or rehearsal of information). In the last experiments we thus sought to further ascertain the role of valence on spatial working memory. This was achieved by presenting emotional pictures with different valence (neutral vs. negative; neutral vs. positive; positive vs. negative) and a similar level of arousal into a single encoding trial.

Findings from Experiment 3 overall show that when arousal is kept constant (at a low level) the position of negative-related objects (Experiment 3A) or positive-related object (Experiment 3B) are better relocated than neutral ones in an immediate VSSP working memory test.

Differently, when both positive and negative stimuli are encoded within the same trial (Experiment 4), there is no effect of emotional valence on spatial performance. In the delayed free recall test, both negative and positive pictures were better remembered than neutral pictures of previous experiments, further supporting the hypothesis that arousal is mainly involved in long-term memory formation. The condition in which both negative and positive pictures are presented but neutral ones are completely absent mimics a condition in which in fact there is no competition since all pictures have a valence. The latter interpretation is supported by the fact that the displacement error of neutral-related objects in Experiment 1A (no competition) is similar to the displacement error of both positive- and negative-related objects in Experiment 4. A possible explanation of our results is that competition for accessing the working memory system could depend upon a dichotomic evaluation between what is endowed with an emotional valence and what is not (emotional vs. non-emotional), independently from the “*valence direction*” (negative or positive). Recently, Willinger et al. (2019) performed an fMRI analysis during an emotional face-matching task and found that the processing of valence directly induces changes in the strength of the bidirectional coupling within a prefrontal-amygdala circuitry (Willinger et al., 2019). Moreover, Kensinger and Schacter (2006) found that independent brain networks specifically processed either positive or negative stimuli. In particular, the ventro-lateral PFC seemed to respond to negative items, whereas the ventro-medial PFC was more engaged during the processing of positive pictures (Kensinger and Schacter, 2006).

Coming back to our results, it is possible to envisage that valenced stimuli, in comparison with neutral stimuli, increase the coupling strength between PFC and limbic structures, favoring memories for valenced information. In this circuit, networks involving the connection between different subregions of PFC and limbic structures may separately process positive and negative valenced stimuli, preventing competition and favoring memory for both negative and positive information. Future experiments should be specifically planned to investigate this issue.

A number of limitations in the present study have to be considered. First, the task used in the present study relied mainly on memory for spatial location and did not require to assign specific objects to these locations: since all to-be-relocated stimuli were rectangles, participants did not have to assign specific objects to spatial locations. It might be interesting to analyze in further experiments the effect of emotions on object-to-position memory. Moreover, different encoding procedures and experimental material should be considered in future experiments. For example, the effects of incidental vs. intentional encoding of the emotional pictures should be compared in order to verify if the advantage due to the competition effect is independent from the encoding procedure. Indeed, a positive correlation between the release of an arousal-related hormone and long-term memory for emotional pictures has been found with intentional, but not with incidental encoding (Preuß et al., 2009). It is interesting to note, however, that no differences due to encoding procedures were found for spatial working memory (D’Argembeau and Van der Linden, 2004).

## Conclusion

In conclusion, the results of the present study suggest that the interplay between arousal and valence is crucial in driving information processing into the memory systems. In this interplay, we hypothesized a differential role of valence and arousal in memory processing. Arousal could act in a relatively automatic way, already from the early stages of perception and perhaps prioritizing the access to the memory system. Moreover, information about the arousal level of learned material can contribute to the long-term memory consolidation process, by the release of arousal-related hormones (i.e., cortisol). In the meanwhile, emotionally valenced stimuli could drive the competition between stimulus representations in the working memory system at a more explicit level. Therefore, within-object features (such as spatial location) of emotionally valenced stimuli (either positive or negative) could be better processed then those of neutral stimuli, and VSWM performance would be enhanced.

## Data Availability Statement

The datasets generated for this study are available on request to the corresponding author.

## Ethics Statement

The studies involving human participants were carried out according to the recommendations of the Committees for Ethics, Department of Psychology Sapienza, University of Rome, and of CERS, LUMSA University. The participants provided their written informed consent to participate in this study.

## Author Contributions

MC developed the idea for this study and drafted the manuscript. MC, DS, CR-A, and VC contributed to the conception and designed the study. BC collected the data and organized the database. MC, CR-A, DS, and VC analyzed and interpreted the data. SL and FD contributed to the discussion of content-related issues and critical revision of the manuscript, and wrote sections of the manuscript. MC and CR-A wrote the final version of the manuscript. All authors contributed to the manuscript revision, read, and approved the submitted version.

## Conflict of Interest

The authors declare that the research was conducted in the absence of any commercial or financial relationships that could be construed as a potential conflict of interest.

## Appendix

### Experiment 1

#### Experiment 1A

Negative IAPS pictures: # 1220, 6244, 9427, 9630, 6555, 9042, 9495, 9611

Neutral IAPS pictures: # 7035, 7060, 7110, 7491, 7490, 2480, 7700, 7234

#### Experiment 1B

Negative IAPS pictures: # 1220, 6244, 9427, 9630

Neutral IAPS pictures: # 7035, 7060, 7110, 7491

#### Experiment 1C

Positive IAPS pictures: # 1710, 7270, 8190, 8500

Neutral IAPS pictures: # 7035, 7060, 7110, 7491

### Experiment 2

Negative IAPS pictures High arousal: #1050, 9940, 6550, 6230

Negative IAPS pictures low arousal: #1220, 6244, 9427, 9630

Neutral IAPS pictures: #7175, 7010, 7006, 7950 and #7035, 7060, 7110, 7491

### Experiment 3

#### Experiment 3A

Negative IAPS pictures: #2722, 2590, 7078, 9220

Neutral IAPS pictures: #2214, 2484, 7011, 7290

#### Experiment 3B

Positive IAPS pictures: #1441, 2091, 2540, 5780

Neutral IAPS pictures: #2214, 2484, 7011, 7290

### Experiment 4

Negative IAPS pictures: #1220, 6244, 9427, 9630

Positive IAPS pictures: #1710, 5480, 5833, 8470

## Supplementary Material

FIGURE S1 Mean displacement error (pixel) in a re-location task in which object-positions were tagged by negative (black square) or neutral (white square) IAPS pictures, and in a task (performed 3 hours later) in which object-positions were tagged by pictures built by scrambling pixels of different colors (Control). Bars: standard error mean.
