# Supplementary figures and images for: The Effect of Emotional Valence and Arousal on Visuo-Spatial Working Memory: Incidental Emotional Learning and Memory for Object-Location
Source: Front Psychol. 2019 Nov 19;10:2587. doi: 10.3389/fpsyg.2019.02587 (PMC6877739; doi:10.3389/fpsyg.2019.02587)

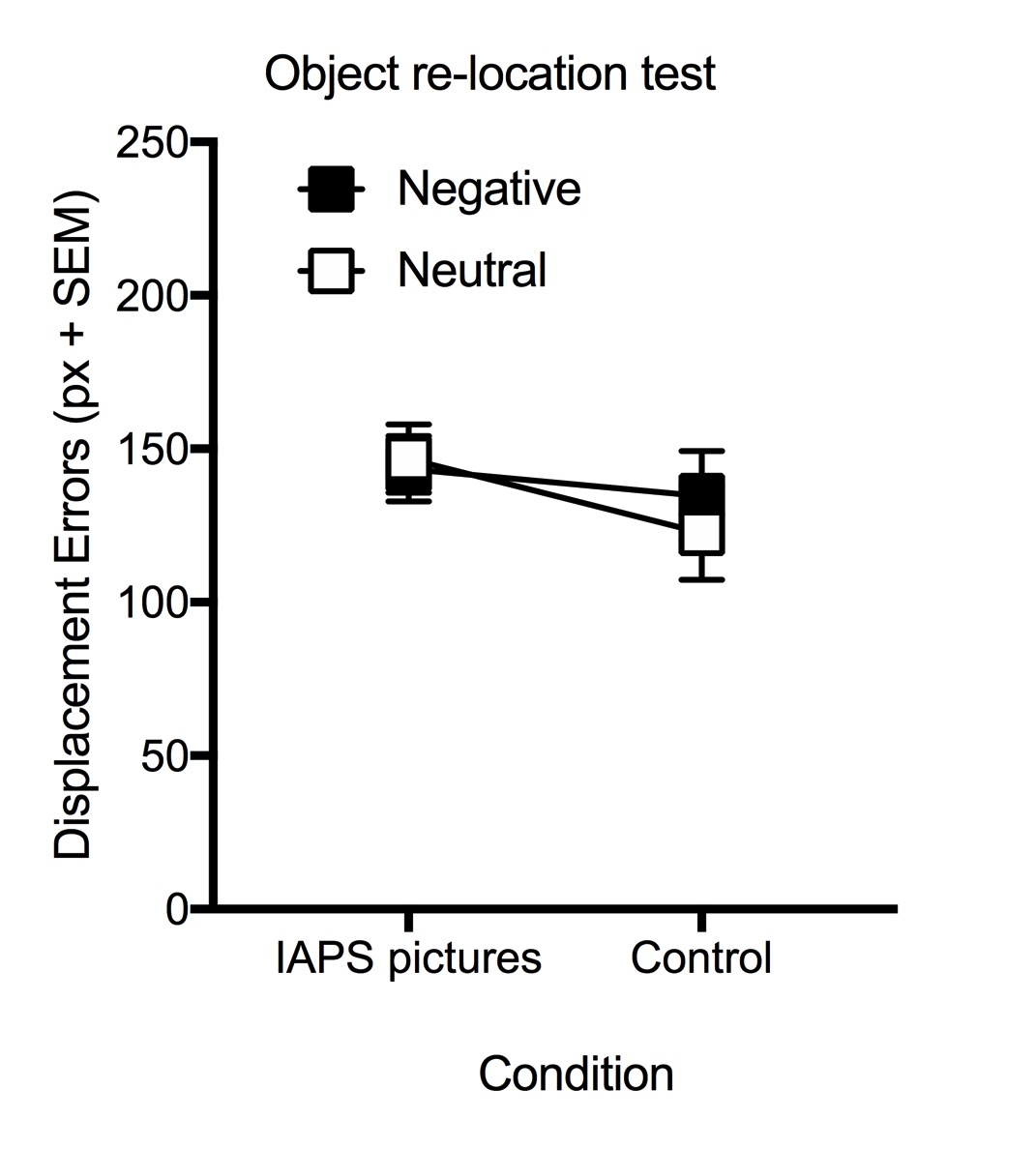

Supplement: FIGURE S1 — Mean displacement error (pixel) in a re-location task in which object-positions were tagged by negative (black square) or neutral (white square) IAPS pictures, and in a task (performed 3 hours later) in which object-positions were tagged by pictures built by scrambling pixels of different colors (Control). Bars: standard error mean. [file Image_1.JPEG]
